# A 10-Bit 300 kS/s Reference-Voltage Regulator Free SAR ADC for Wireless-Powered Implantable Medical Devices

**DOI:** 10.3390/s18072131

**Published:** 2018-07-03

**Authors:** Yongkui Yang, Jun Zhou, Xin Liu, Wang Ling Goh

**Affiliations:** 1 School of Electrical and Electronic Engineering, Nanyang Technological University, Singapore 639798, Singapore; yyang014@e.ntu.edu.sg (Y.Y.); liuxin@ieee.org (X.L.); ewlgoh@ntu.edu.sg (W.L.G.); 2 School of Information and Communication Engineering, University of Electronic Science and Technology of China, Chengdu 611731, China

**Keywords:** SAR ADC, low power, reference-voltage regulator free, implantable, medical device

## Abstract

This paper presents a reference-voltage regulator free successive-approximation-register analog-to-digital converters (SAR ADC) with self-timed pre-charging for wireless-powered implantable medical devices. Assisted by a self-timed pre-charging technique, the proposed SAR ADC eliminates the need for a power-hungry reference-voltage regulator and area-consuming decoupling capacitor while maintaining insensitivity to the supply voltage fluctuation. Fabricated with a 0.18-µm complementary metal–oxide–semiconductor (CMOS) technology, the proposed SAR ADC achieves a Signal To Noise And Distortion Ratio (SNDR) of 53.32 dB operating at 0.8 V with a supply voltage fluctuation of 50 mV_pp_ and consumes a total power of 2.72 µW at a sampling rate of 300 kS/s. Including the self-timed pre-charging circuits, the total figure-of-merit (FOM) is 23.9 fJ/conversion-step and the total area occupied is 0.105 mm^2^.

## 1. Introduction

**Figure 1:**
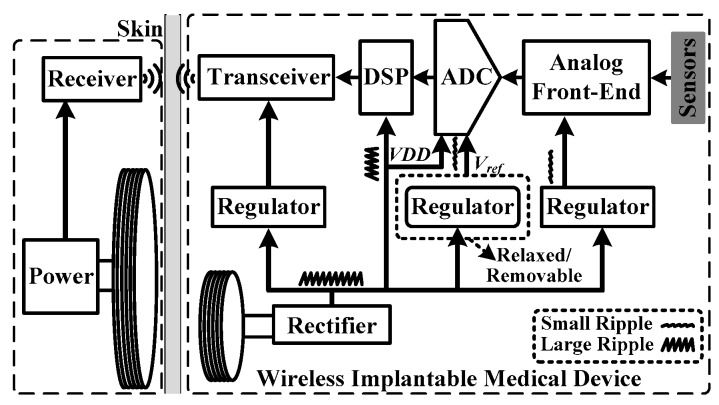
Block diagram of a typical wireless implantable medical device.

As one of the typical health monitoring applications, wireless implantable medical devices are powered via wireless power transfer, which is shown in Figure 1. It is mandatory to include a rectifier to convert the coupled AC power into DC power. Even with advanced rectifiers, such as the reconfigurable resonant regulating rectifier (R^3^ rectifier), the ripple of the rectified voltage can still be measured in tens of mV [1]. For conventional SAR ADCs adopted in such systems [2,3,4,5,6,7,8,9], a dedicated regulator is needed to stabilize the reference voltage against the ADC core. However, the regulator consumes significant power [2,3,4]. Besides, decoupling capacitors of a few hundred pF or larger values are needed to maintain the stable operation of the regulator [10]. As it is preferred to have decoupling capacitors on chips in wireless implantable medical devices for system miniaturization, the increase in silicon area and hence, cost are inevitable.

In the past, most designs focused on optimizing the power and area of the sub-blocks in the SAR ADC core [5,6,7,8], while only a few studies aimed to optimize the reference-voltage regulator [2,3,4,11]. It was found that if the power consumption of the reference-voltage regulator is included, the FOM of ADC degrades from 100 fJ/c-s to 1000 fJ/c-s [2] or from 25 fJ/c-s to 738 fJ/c-s [3]. In a previous study [4], a SAR ADC with energy-efficient reference generation was proposed, in which power saving was realized by applying a duty-cycle technique in the reference generator. However, an always-on low dropout regulator is needed to drive the ADC core, which leads to quiescent current and dropout voltage. The power dissipated by the regulator accounts for more than 30% of the total power consumption of the SAR ADC. Besides, a large decoupling capacitor is needed to maintain the stable operation of the regulator.

In this paper, we present a reference-voltage regulator free SAR ADC using self-timed pre-charging for wireless-powered implantable medical devices. It eliminates the power-hungry reference-voltage regulator to reduce the overall power consumption of the SAR ADC. Furthermore, the need for a large on-chip decoupling capacitor is eliminated, which helps to reduce the silicon area. The proposed design achieves good SNDR under the conditions of supply voltage fluctuation. Taking into account the power and area of the reference-voltage regulator, the proposed SAR ADC achieves better FOM with a small area compared to several existing SAR ADC designs.

## 2. Proposed Reference-Voltage Regulator Free SAR ADC

In SAR ADC, any disturbance or error in the reference voltage will cause the digitized output codes to deviate from the correct value with errors. Consequently, it introduces large amounts of noise and decreases the SNDR of the ADC. As discussed previously, in wireless-powered implantable medical devices, the output voltage of the rectifier contains large ripples and cannot be used directly as the reference voltage in conventional SAR ADC. Therefore, in the typical wireless-powered implantable medical devices, a dedicated regulator with decoupling capacitor is used to provide a stable reference voltage for charging the digital-to-analog converter (DAC) capacitors, which dramatically increases the power consumption and required area. 

### 2.1. System Architecture

**Figure 2:**
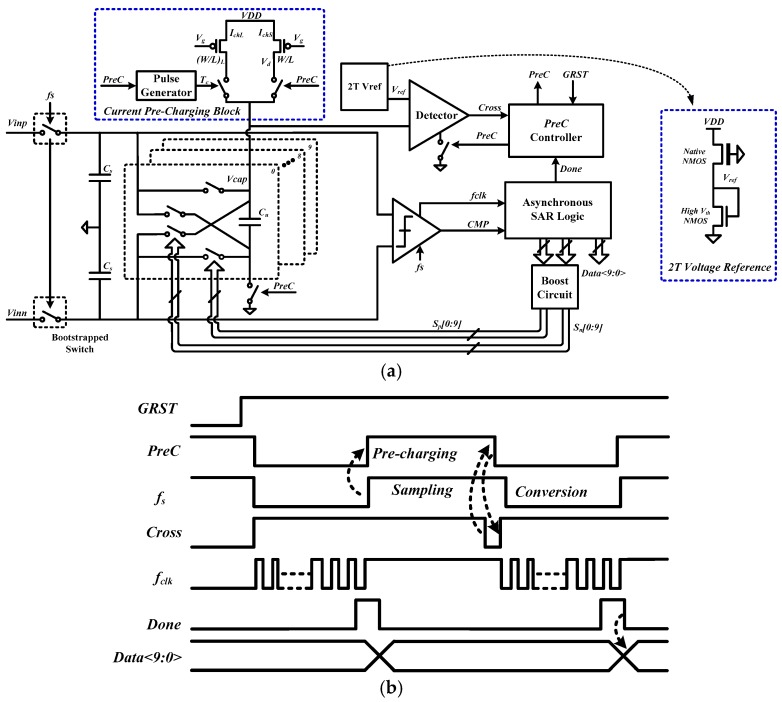
(**a**) Architecture and (**b**) timing diagram of the proposed reference-voltage regulator free SAR ADC with self-timed pre-charging.

In order to eliminate the need for the reference-voltage regulator and the decoupling capacitor, we propose a SAR ADC with self-timed pre-charging, which is shown in Figure 2. It consists of a current pre-charging circuit, a charge detector and a 10-bit conventional charge-sharing SAR ADC [9] using asynchronous SAR logic. The most significant bit (MSB) uses 32-unit capacitors, while the 5 least significant bits (LSBs) use 1 unit capacitor each. The rising edge of *f_s_* will trigger the *PreC* signal to increase in order to perform pre-charging. When the voltage crosses the *V_ref_*, a Cross signal is generated by the detector and it will pull down the *PreC* signal to end the pre-charging. Furthermore, the logic low of *PreC* will set the Cross to high logic. The internal clock *f_clk_* for asynchronous SAR logic is generated by the comparator [4].

**Figure 3:**
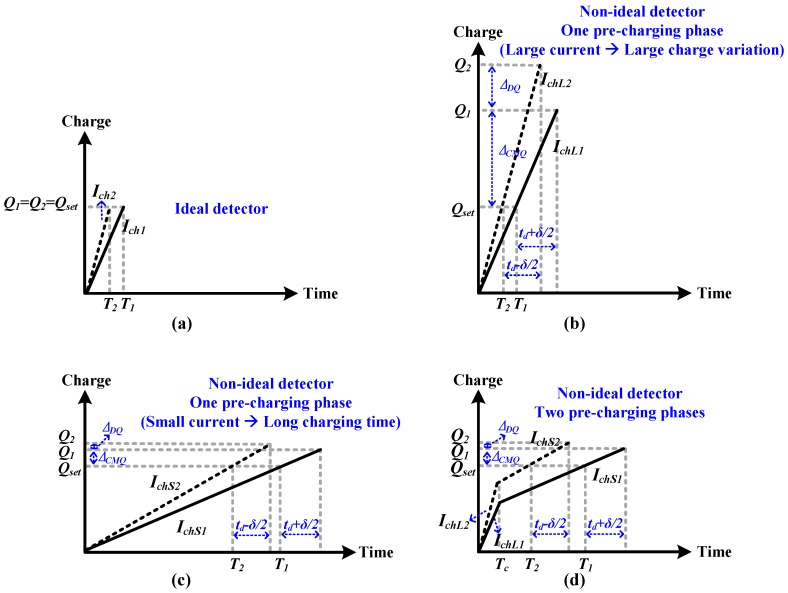
Self-timed pre-charging (**a**) ideal detector; (**b**) non-ideal detector using one pre-charging phase with large current; (**c**) non-ideal detector using one pre-charging phase with small current; and (**d**) non-ideal detector using two pre-charging phases.

During the sampling period, the input signal is sampled onto the sampling capacitors *C_s_*. At the same time, the DAC capacitors are pre-charged with a current source. Controlled by a charge detector, the current source will be turned off when the charge reaches a pre-set value *Q_set_*, which is called self-timed pre-charging. During the conversion period, according to the results from the comparator, the charge in the DAC capacitors is added to or subtracted from *C_s_*. Therefore, if the *V_ref_* fluctuates during sampling periods, the charge in the DAC capacitors will also fluctuate. The common mode part of charge variation may cause an error, but it can be removed by calibration, which also occurs in conventional ADCs. However, the differential part is not constant and cannot be removed by calibration. This will cause an error and SNDR degradation if the differential part is larger than the LSB charge (2.5 fC in this design). Figure 3a shows the pre-charging process with different charging currents (i.e., *I_ch_*_1_ and *I_ch_*_2_). In Figure 3a, we assume that the charge detector is ideal with the response time being zero. Although *I_ch_*_1_ and *I_ch_*_2_ are different, the same charge is obtained through different charging times (i.e., *T*_1_ and *T*_2_). However, in practice, the charge detector has a response time or delay. For wireless-powered medical devices, the delay is affected by the supply voltage fluctuation. As illustrated in Figure 3b, with a small difference in the supply voltage, the delay will be *t_d_* + *δ*/2 and *t_d_* − *δ*/2, respectively, where *t_d_* is the basic delay of the charge detector and *δ* is a delay difference caused by the supply voltage fluctuation. The delay difference induces a difference in the charge (i.e., ∆*_Q_*), which can be expressed as:

(1) $$\begin{array}{cc} \Delta_{Q}=Q_{2}-Q_{1} & =\int_{0}^{t_{d}-\delta /2}I_{ch2}\times t-\int_{0}^{t_{d}+\delta /2}I_{ch1}\times t\\ & =\Delta_{CMQ}+\Delta_{DQ}, \end{array}$$

where ∆*_CMQ_* is the common part of charge variation and ∆*_DQ_* is the differential part. As mentioned previously, the common part of charge variation may cause an error but it can be removed by calibration, which also occurs in conventional ADC. However, the differential part is not constant and cannot be removed by calibration. In order to not affect the performance, the differential part ∆*_DQ_* needs to be less than the LSB charge in the design. For the pre-charging scheme, a large pre-charging current is used to shorten the pre-charging time to achieve a sufficient sampling rate. However, this larger current is more sensitive to supply voltage fluctuation, thus causing large charge variation, which is shown in Figure 3b. This can be explained as follows. With a current source as shown in Figure 2, the charging current (i.e., *I_ch_*) can be expressed as:

(2) $$I_{ch}=\frac{KW}{2L}{\left(VDD-V_{g}-V_{th}\right)}^{2}(\mathrm{active}\;\mathrm{region}),$$

(3) $$I_{ch}=\frac{KW}{L}\left[\left(VDD-V_{g}-V_{th}\right)\times \left(VDD-V_{d}\right)-\frac{{\left(VDD-V_{d}\right)}^{2}}{2}\right](\mathrm{triode}\;\mathrm{region}),$$

where *K* and *V_th_* are the transconductance parameter and threshold voltage of the PMOS, respectively. To determine *I_ch_*’s sensitivity to *VDD*, the derivative of *I_ch_* is obtained with respect to *VDD* in Equations (2) and (3). This gives:

(4) $$\frac{\partial I_{ch}}{\partial VDD}=\frac{KW}{L}\left(VDD-V_{g}-V_{th}\right).$$

From Equation (4), it can be seen that with a smaller (*VDD*−*V_g_*−*V_th_*) or current *I_ch_*, the current’s sensitivity to *VDD* is smaller, which results in a smaller charge variation (see Figure 3c). However, this smaller pre-charging current needs longer charging time and this limits the sampling rate of the ADC.

To achieve a sufficient sampling rate while ensuring a small charge variation (less than 1 LSB charge), we proposed a pre-charging scheme with two charging phases, which is shown in Figure 3d. During the first charging phase, the DAC capacitors are quickly charged with a large charging current (i.e., *I_chL_*) and constant charging time (i.e., *T_c_*). In the second charging phase, a small charging current (i.e., *I_chS_*) is used to complete the rest of charging and is turned off when the charge reaches the pre-set value *Q_set_*. By using the two-phase pre-charging scheme, the charge variation is reduced and the charging time is also shortened.

### 2.2. Circuit Implementation Details

To save power consumption, a 2-transistor (2T) voltage reference [12] is adopted. A native N-channel metal oxide semiconductor FET (NMOS) and a high threshold NMOS are used to generate a *V_ref_* with 400 mV, which is shown in Figure 2. The value of the sampling capacitor *C_s_* used is 3.2 pF. With a maximum signal swing of roughly 800 mV peak-to-peak differential, the *LSB charge* of this ADC can be calculated as:

(5) $$LSB\;charge\;=\frac{V_{in}C_{s}}{2^{10}}\approx 2.5fC.$$

**Figure 4:**
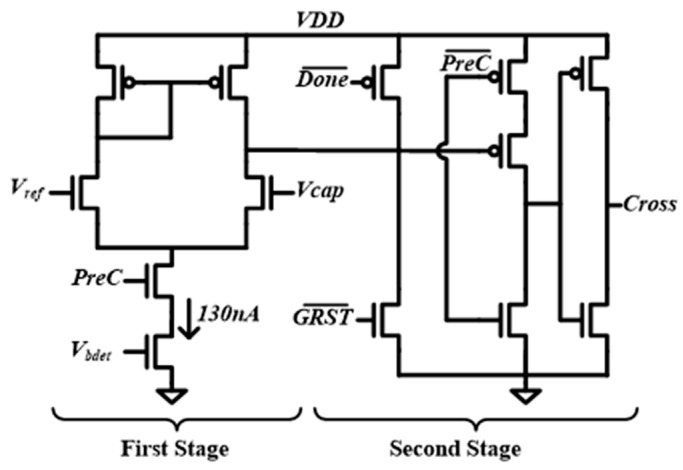
Schematic of the proposed charge detector.

Figure 4 shows the schematic of the charge detector used for controlling the self-timed pre-charging. It consists of two stages. The first stage amplifies the input signals and converts the voltage difference between *V_cap_* and *V_ref_* to a single-ended output. The output of the first stage will trigger the second stage when *V_cap_* crosses *V_ref_*. To minimize the power consumption, the first stage operates only during the pre-charging phase, which occurs when *PreC* is actively high. The simulation shows that the delay of the charge detector varies from 418.3 ns to 410.8 ns when the supply voltage is changed from 0.775 V to 0.825 V.

**Figure 5:**
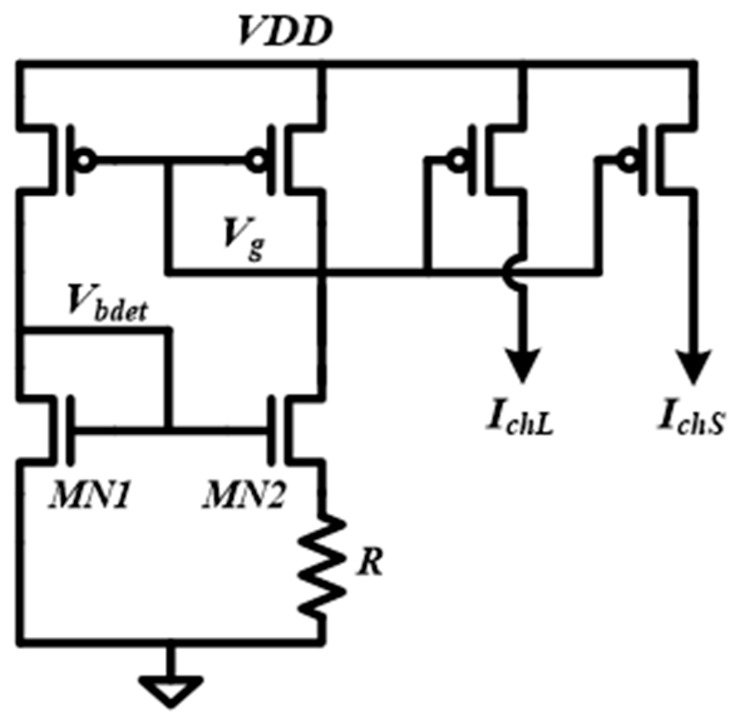
Schematic of the current source used for self-timed pre-charging.

The schematic of the current source used for pre-charging is shown in Figure 5. The reference current, *I_ref_*, is equal to the difference of the threshold voltage between MN1 and MN2 divided by the value of resistor R, which is independent of the supply. The output currents, *I_chL_* and *I_chS_*, are generated from *I_ref_* through the current mirror. The *I_chL_* is used as the first phase charging current and is about 14 µA. The *I_chS_* is used as the second phase charging current and is about 200 nA. The values of *I_chL_* and *I_chS_* are determined through extensive simulation by considering both the charge variation and pre-charging time. The simulation shows that when *VDD* is larger than 0.6 V, the sensitivity of *I_chL_* and *I_chS_* to *VDD* are 19 µA/V and 36 nA/V, respectively. With a smaller current, the sensitivity is much lower, which verified the previous analysis presented in Section 2.1. To save power and area, the beta-multiplier reference also generates *V_bdet_* for the first stage amplifier in the charge detector.

The simulation also shows that the *I_chS_* varies from 203.9 nA to 205.7 nA when the supply voltage varies from 0.775 V to 0.825 V. Using these values together with the delay variation of charge detector (from 418.3 ns to 410.8 ns), the charge variation can be calculated from Equation (1). The calculated common part of the charge variation is 84.9 fC. Even though the common part is large, as discussed previously, it only causes a gain error and the error can be easily removed through calibration. Due to the implementation of the two-phase pre-charging scheme, the differential part is only 0.4 fC, which is negligible when compared to the 1-LSB charge (~2.5 fC). The simulation shows that the two-phase pre-charging time varies from 1.1 µs to 2.3 µs when the supply voltage varies from 0.9 V to 0.7 V. This pre-charging time is sufficiently short for a 300 kS/s sampling rate.

## 3. Measurement Results

**Figure 6:**
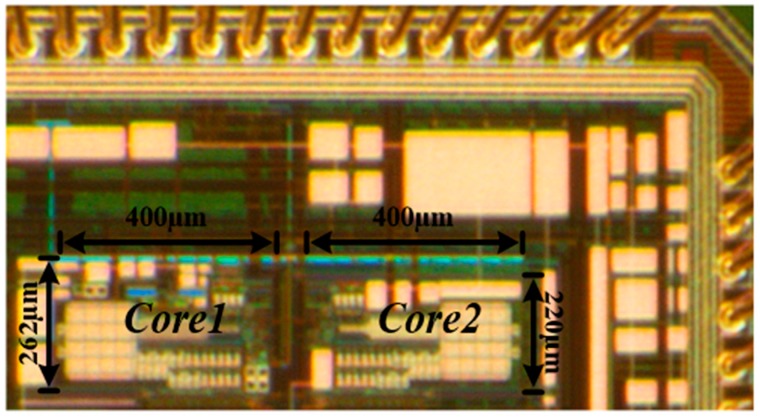
Die photo of two SAR ADCs where Core1 is the proposed design and Core2 is the conventional design.

The proposed reference-voltage regulator free SAR ADC has been implemented and fabricated with a 0.18-µm CMOS technology. The chip micrograph is shown in Figure 6. Core1 is the proposed SAR ADC with an area of 0.105 mm^2^. Core2 is the conventional SAR ADC where all the circuits are the same as Core1 except that the self-timed pre-charging circuits are not included and the reference voltage is directly obtained from an external supply. Core2 occupies 0.088 mm^2^.

**Figure 7:**
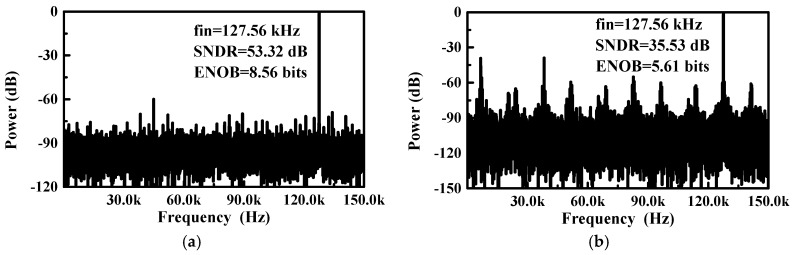
Measured output spectrum operating at 300 kS/s when a sinusoidal fluctuation of 50 mV_pp_ and 134.2 kHz is added to the 0.8 V power supply of (**a**) proposed SAR ADC (i.e., Core1) and (**b**) conventional SAR ADC (i.e., Core2).

**Figure 8:**
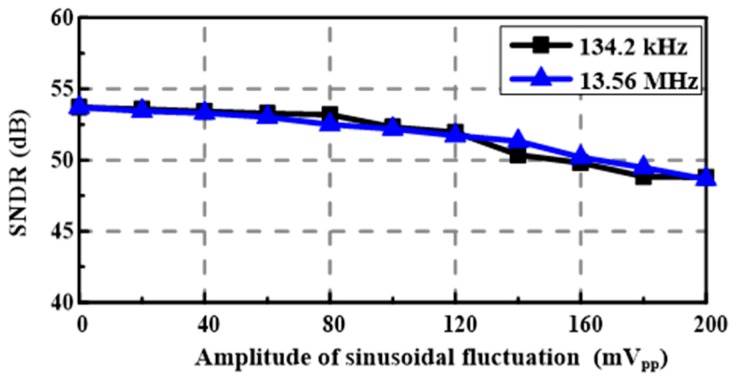
The SNDR versus the amplitude of sinusoidal fluctuation added to the 0.8 V power supply.

**Figure 9:**
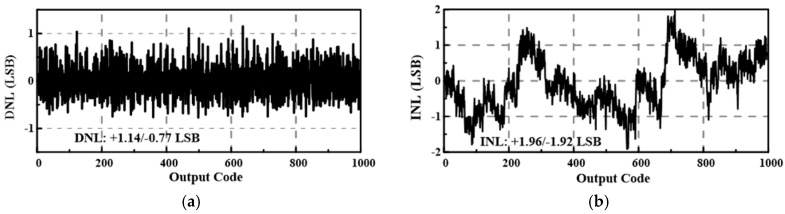
The measured (**a**) DNL and (**b**) INL of the proposed ADC when a sinusoidal fluctuation of 50 mV_pp_ is added to the 0.8 V power supply.

The proposed SAR ADC uses a single power supply of 0.8 V. To demonstrate its insensitivity to supply voltage fluctuation, a sinusoidal fluctuation with an amplitude range of 0–200 mV_pp_ is added to the supply voltage. The frequencies of the sinusoidal fluctuation are set to 134.2 kHz and 13.56 MHz, which are widely used carrier frequencies in wireless power transfer [1]. Figure 7 shows a 32,768-point fast Fourier transform (FFT) plot for a 127.56-kHz input signal sampled at 300 kS/s under a sinusoidal fluctuation of 50 mV_pp_ and 134.2 kHz. The corresponding effective number of bits (ENOB) of the proposed SAR ADC (i.e., Core1) and conventional SAR ADC (i.e., Core2) are 8.56 bits and 5.61 bits, respectively. Figure 8 shows the SNDR versus the amplitude of the sinusoidal fluctuation. When the amplitude of sinusoidal fluctuation increases from 0 to 200 mV_pp_, the SNDR decreases by 4.91 dB and 5.05 dB for sinusoidal fluctuations of 134.2 kHz and 13.56 MHz, respectively. As indicated in Figure 9, the measured differential non linearity (DNL) and integral non linearity (INL) are +1.14/−0.77 LSB and +1.96/−1.92 LSB, respectively.

The measured total power consumption of the proposed SAR ADC (i.e., Core1), including the self-timed pre-charging circuits, is 2.72 µW at 300 kS/s and the corresponding FOM is 23.9 fJ/c-s. If the gain error is calibrated on the chip, the FOM will be increased by about 25% due to the power consumed by the multiplier (power consumption estimated based on reference [13]). Under the same testing conditions, the measured power consumption of the conventional SAR ADC (i.e., Core2) is 2.52 µW. Therefore, the power dissipation of the self-timed pre-charging circuits is around 0.2 µW. This accounts for only 7.3% of the total power consumption of ADC, while a reference-voltage regulator typically consumes at least 30% of [4] or even several times [2,3] the power consumption of ADC.

The comparisons between the proposed SAR ADC and other existing SAR ADCs fabricated with similar technology nodes are provided in Table 1. With a supply voltage fluctuation of 50 mV_pp_, the proposed design has a similar SNDR to other designs. We noted that the power consumption of the ADC core reported in references [5,6,7,8] do not consider the reference-voltage regulator. Hence, for fair comparison, we provided the estimated power consumed in these reference-voltage regulators in Table 1. The quiescent current of the regulators are estimated using the regulator *FOM* formula (i.e., *FOM_reg_*), which was defined in [10] as:

(6) IQ=CdFOMreg×Iref2Δvout=1.43μFns×Iref2Δvout,

where *C_d_* is the decoupling capacitor, ∆*_vout_* is 1 LSB, *I_Q_* is the regulators’ quiescent current and *I_ref_* is the current used to charge DAC capacitors. Assuming that all the designs have the same *C_d_* and *FOM_reg_* value as in reference [4] (i.e., *C_d_*/*FOM_reg_* = 1.43 µF/ns), the quiescent current of the reference-voltage regulator for each design can be calculated using the above formula. Besides, we assume a dropout voltage of 200 mV as calculated in reference [4]. Based on the quiescent current and the dropout voltage, the total power consumption of regulator for each design can be calculated as:

(7) $$P_{Vref\;reg.}=I_{Q}\times VDD+I_{ref}\times V_{dropout}$$

As shown in Table 1, the proposed self-timed pre-charging SAR ADC achieves a better FOM than the other designs when taking into account the reference-voltage regulator. Including the self-timed pre-charging circuits, the area of the proposed design is similar to that of the other designs without the reference-voltage regulator. Moreover, the decoupling capacitor is not required in our design, which helps to further reduce the total area and cost. Without the reference-voltage regulator, our design finally achieves an ENOB of 8.56 bits, which is adequate for medical devices [5,6,8].

## 4. Conclusions

In this paper, a SAR ADC with self-timed pre-charging is proposed to eliminate the reference-voltage regulator and decoupling capacitor for wireless-powered implantable medical devices as they dramatically increase power consumption and required area. Fabricated with a 0.18-µm CMOS technology, the proposed self-timed pre-charging SAR ADC achieves a SNDR of 53.32 dB at 0.8 V with a supply voltage fluctuation of 50 mV_pp_ while consuming a total power of 2.72 µW at a sampling rate of 300 kS/s. Including the self-timed pre-charging circuits, the total FOM is 23.9 fJ/c-s and the total area is 0.105 mm^2^.

## Figures and Tables

**Table 1:** Performance comparison.

	[5]	[6]	[7]	[8]	This Work
Tech. (µm)	0.18	0.18	0.13	0.18	0.18
Area (mm^2^)	-	0.151 (w/o Reg.)	0.872 (w/o Reg.)	0.118 (w/o Reg.)	0.088 (w/STP *) 0.105 (w/STP)
Supply (V)	0.45	0.9	0.5/1	1	0.8
Resolution	9	9	10	10	10
fs (kS/s)	200	100	1100	100	300
SNDR (dB)	51.54	50.1	54.6	58.83	53.32 w/50 mV_pp_ Ripple
Power w/o *V_ref_* Reg. (µW)	1.35	1.33	15.6	1.72	2.52 w/o STP
FOM w/o *V_ref_* Reg. (fJ/c-s)	22.0	51.3	31.8	24.1	22.1 w/o STP
*I_ref_* (µA)	0.73	0.69	8.4	0.42	-
∆*_vout_* (mV)	0.87	1.75	1.95	0.97	-
*I_Q_* (µA)	0.87	0.38	51.84	0.25	-
*V_dropout_* (mV)	200	200	200	200	-
P*_Vref_* Reg. (µW) **	0.54	0.49	53.5	0.34	0
Power w/*V_ref_* Reg. (µW)	1.89	1.81	69.1	2.06	2.72 w/STP
FOM w/*V_ref_* Reg. (fJ/c-s)	30.8	70.1	140.9	28.7	23.9 w/STP

* STP stands for self-timed pre-charging. ** Power consumption of reference-voltage regulators needed in references [5,6,7,8] are estimated based on the regulator used in reference [4] with the assumption that they have the same regulator *FOM_reg_* defined in reference [10] and the same dropout voltage.
